# Supplementary material for: ARHGEF18/p114RhoGEF Coordinates PKA/CREB Signaling and Actomyosin Remodeling to Promote Trophoblast Cell-Cell Fusion During Placenta Morphogenesis
Source: Front Cell Dev Biol. 2021 Mar 25;9:658006. doi: 10.3389/fcell.2021.658006 (PMC8027320; doi:10.3389/fcell.2021.658006)
Supplement: Supplementary file 1 [file Data_Sheet_1.PDF]

Supplementary Material

SUPPLEMENTARY TABLE

Antibodies	Source	Identifier
E-cadherin, mouse monoclonal	BD Biosciences	610182
Laminin, rabbit polyclonal	Sigma-Aldrich	L9393
MCT4, rabbit polyclonal	Merck Millipore	AB3314P
p114RhoGEF, rabbit polyclonal	GeneTex	GTX102223
p120catenin, goat polyclonal	Santa Cruz Biotechnology	sc-373116
Non-muscle myosin-IIA	Sigma-Aldrich	M8064
pp-MLC Thr18S19, rabbit polyclonal	Cell Signaling Technology	3674
VE-Cadherin, mouse monoclonal	BD Biosciences	555661
PECAM-1/CD31, rat monoclonal	BD Biosciences	550274
JAM-A, rabbit polyclonal	Santa Cruz Biotechnology	sc-25629
JACOP/CGNL1, rabbit polyclonal	abcam	ab204500
β-catenin, sheep polyclonal	abcam	ab65747
Vinculin, mouse monoclonal	Sigma-Aldrich	V9131
AKAP12, mouse monoclonal	Santa Cruz Biotechnology	sc-376740
p-CREB Ser133, rabbit polyclonal	Cell Signaling Technology	9198
Gcm1, rabbit polyclonal	Santa Cruz Biotechnology	sc-69411
p-VASP Ser239, rabbit polyclonal	Cell Signaling Technology	3114
ZO-1, mouse monoclonal	ThermoFisher Scientific	339100
α-smooth muscle actin	Sigma	A5228
ZO-1, rabbit polyclonal	Benais-Pont et al. (2003)	NA
α-tubulin	Kreis (1987)	NA
Alexa488 donkey anti-mouse	Jackson ImmunoResearch	715-545-150
Cy3 donkey anti-rabbit	Jackson ImmunoResearch	711-165-152
Alexa647-labelled donkey anti-goat	Jackson ImmunoResearch	705-605-147
Cy3 labelled donkey anti-mouse	Jackson ImmunoResearch	715-165-150
FITC donkey anti-sheep	Jackson ImmunoResearch	713-095-147
HRP-goat anti-rabbit	Jackson ImmunoResearch	111-035-003
HRP-goat anti-mouse	Jackson ImmunoResearch	115-035-003
IRDye 800CW donkey anti-Mouse	LI-COR	926-32212
IRDye 680LT donkey anti-Rabbit	LI-COR	926-68023

**Chemicals**		
Phalloidin-Atto647	Sigma-Aldrich	65906
Biotin-isolectin BSI-B4	Sigma-Aldrich	L2140
RNAiMAX	ThermoFisher Scientific	13778150
TransIT	Mirus Bio	MIR6000
Prolong Gold antifade reagent	ThermoFisher Scientific	P36930
**Hybridization probes**		
Tpbpa	Professor James Cross	Simmons et al., 2007
Hand1	Professor James Cross	Simmons et al., 2007
Gcm1	Professor James Cross	Simmons et al., 2007
SynA	Professor James Cross	Simmons et al., 2007
p114RhoGEF	ThermoFisher Scientific	ViewRNA System

Supplementary Table S1 | Shown are the sources of antibodies, key reagents and hybridisation probes used.

SUPPLEMENTARY FIGURES

A

Genotypes of born mice

Mating strategy	+/+	+/-	-/-
p114♀+/+ X p114♂+/-	52	47	-
p114♀+/- X p114♂+/+	33	35	-
p114♀+/- X p114♂+/-	12	30	0

B

	+/+	+/-	-/-
E10.5-11.5	27	62	22
Turn defect	3.7%	1.6%	18.2%
Effusion	-	1.6%	13.6%
Yolk sac oedema	7.4%	6.5%	9.1%
Anemia	-	3.2%	4.5%
Resorbed	3.7%	6.5%	4.5%

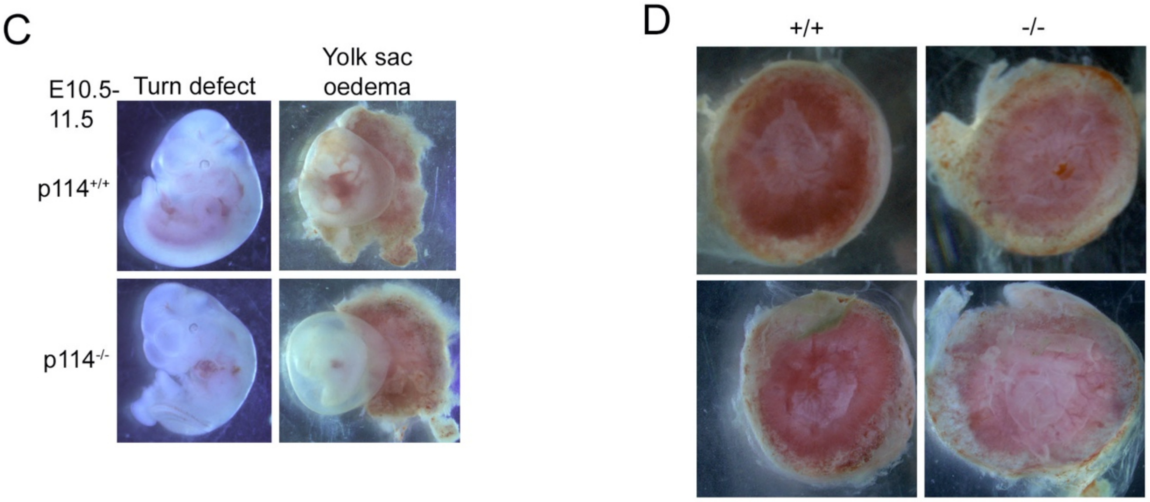

Supplementary Figure 1 | Phenotypic analysis of p114RhoGEF-deficient mice. (**A**) Analysis of litter genotypes derived from inter-crossing of p114$^{+/-}$ with p114$^{+/+}$ or p114$^{+/-}$ mice; no p114$^{-/-}$ mice were born. (**B,C**) Analysis of p114$^{-/-}$ phenotypes at E10.5-11.5 and representative images. (**D**) Representative images of p114$^{-/-}$ placentas at E12.5 suggesting reduced blood content.

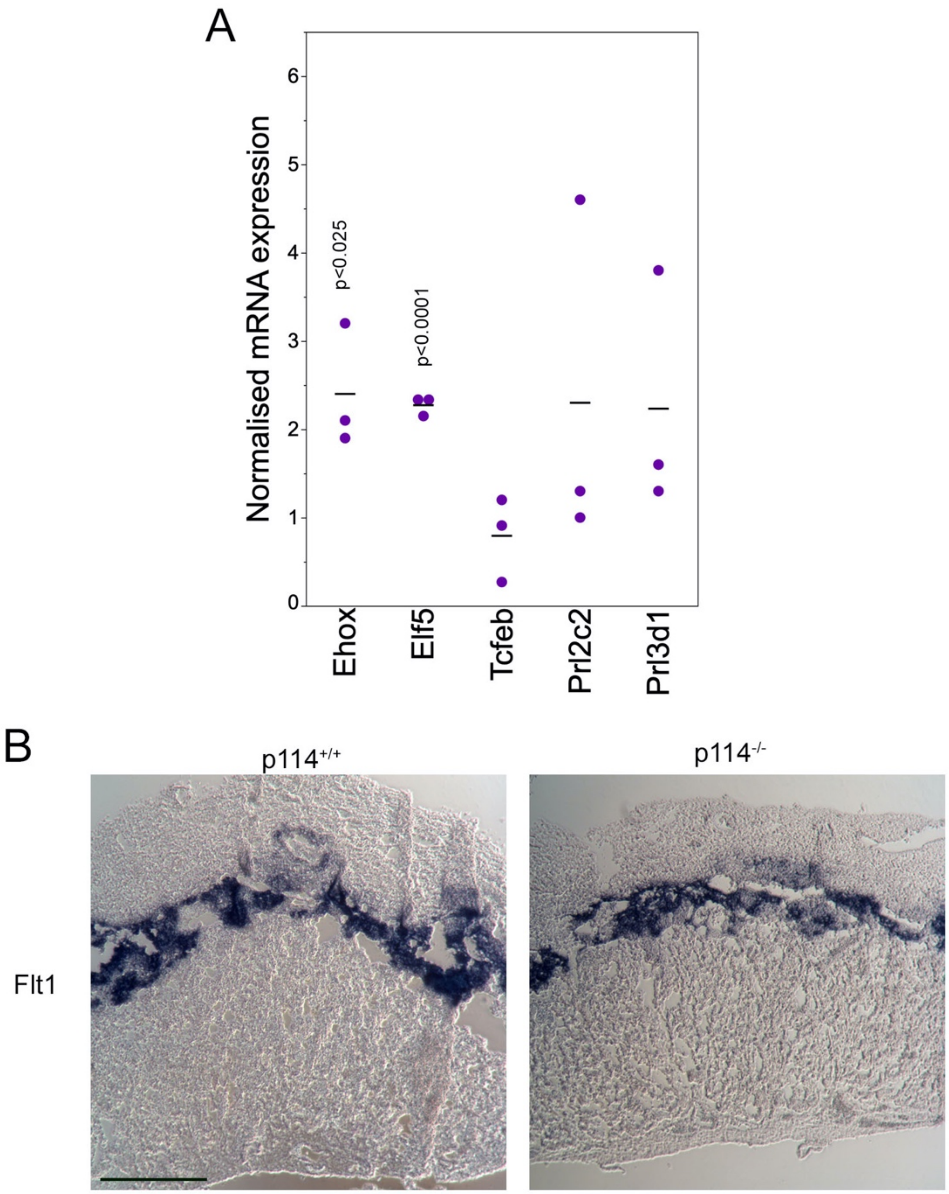

Supplementary Figure 2 | Characterization of TSC and trophoblast marker expression in p114RhoGEF-deficient mice. (**A**) Analysis of RNA expression by RT-qPCR of markers for TSCs and trophoblasts (shown are data points from three experiments, means and p-values derived from t-tests). (**B**) In situ hybridization revealing expression of Flt1 in the junctional zone. Bar: 0.5mm

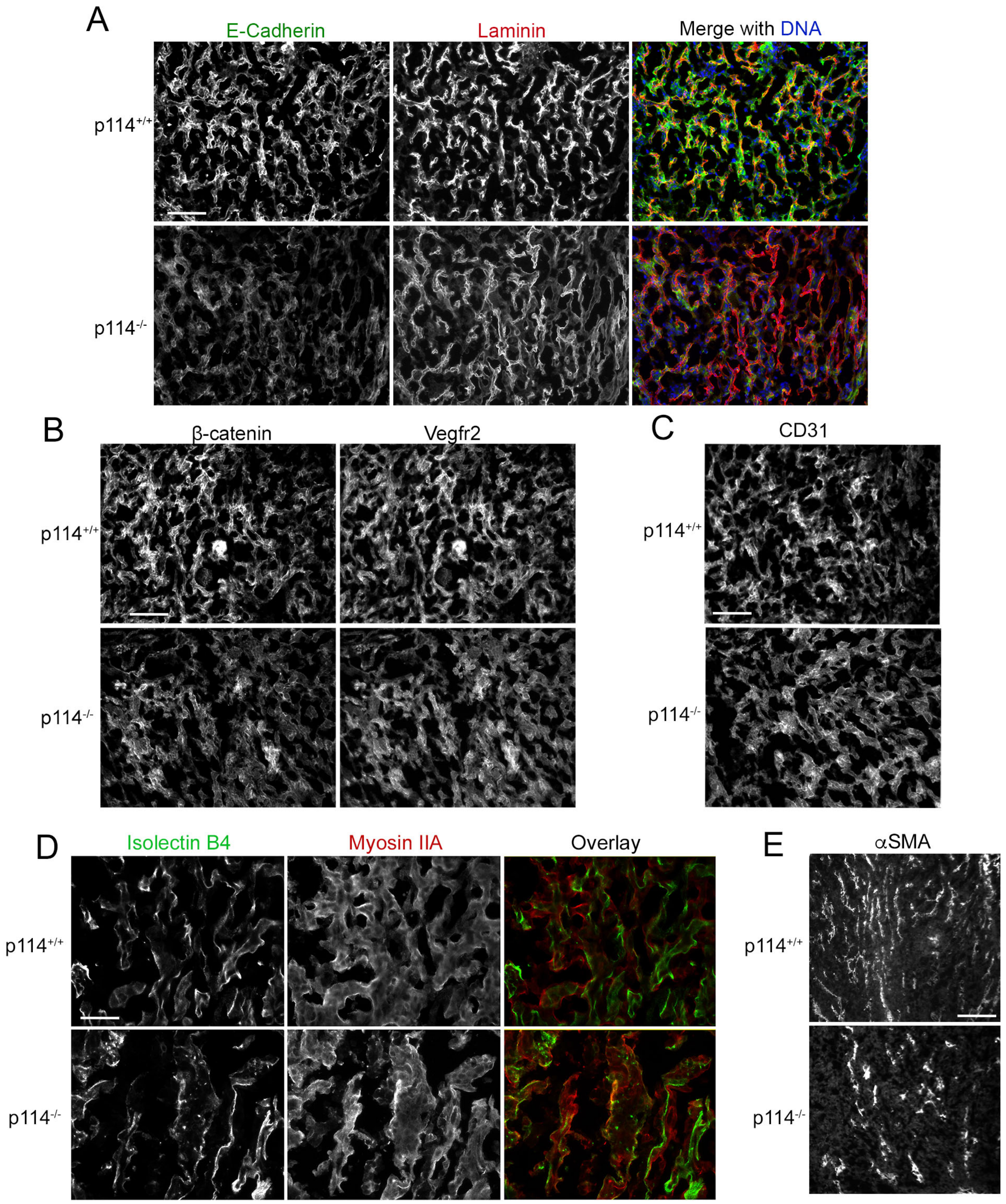

Supplementary Figure 3 | Effect of p114RhoGEF knockout on the expression of proteins of endothelia and syncytiotrophoblasts in the labyrinth layer of the placenta. (**A-C**) Expression of markers for endothelial and syncytiotrophoblasts was analysed by immunofluorescence of cryosections. (**D**) Double staining of Isolectin B4 and myosin II reveals a stronger effect on myosin II organisation in Islolectin-B4-negative (syncytiotrophoblasts) than in positive (endothelial cells) areas. (**E**) Labelling of α-smooth muscle actin by immunofluorescence of placental cryosections. Bars: A-C And E, 200μm; D, 100μm.

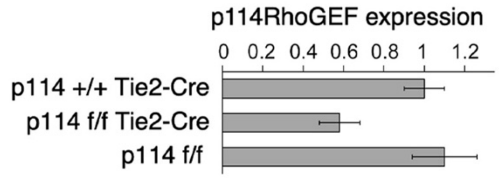

Supplementary Figure 4 | Reduced expression of p114RhoGF in aorta samples of endothelial specific p114RhoGEF knockout mice. Analysis of p114RhoGEF expression by RT-qPCR from total RNA isolated from aorta samples derived from control or endothelial specific knockout mice. Shown are examples of mice analysed.

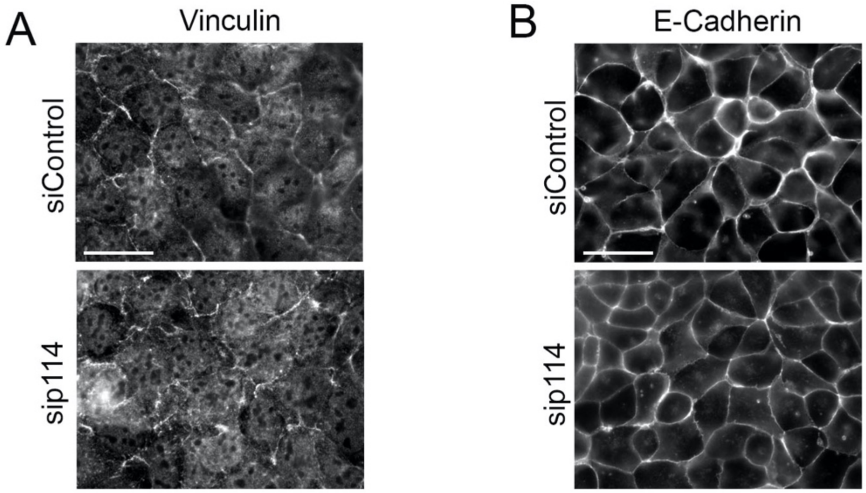

Supplementary Figure 5 | Localization of vinculin and E-cadherin in TSR-26 cells. Effect of p114RhoGEF depletion on vinculin (**A**) and the adherens junction protein E-cadherin (**B**) in TSR-26 cells was analysed by immunofluorescence microscopy. Bars: 40µm.

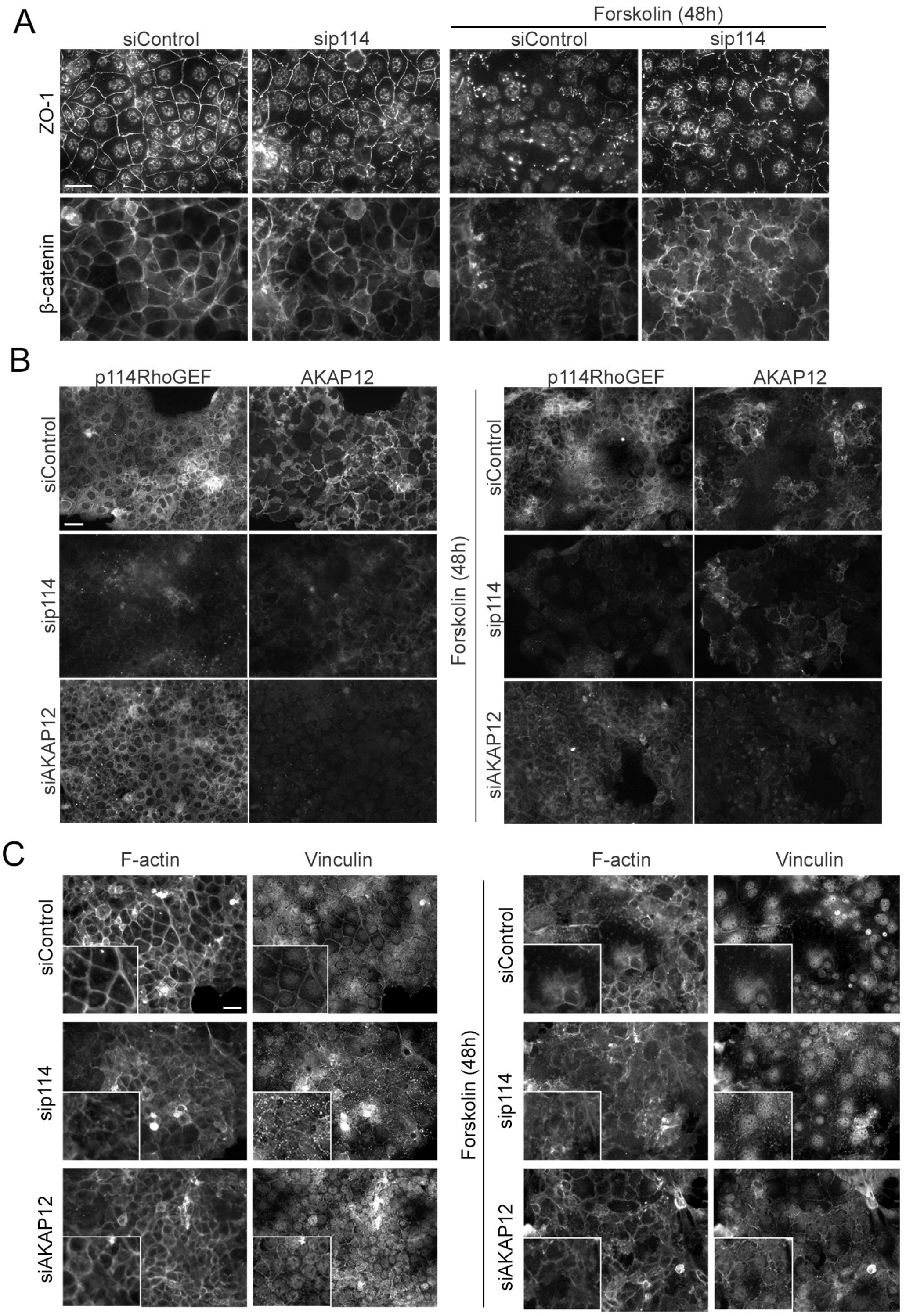

Supplementary Figure 6 | Inhibition of cell-cell fusion of BeWo cells. BeWo cells transfected with control, p114RhoGEF or AKAP12 siRNAs were treated with 100µM forskolin for 48h and then analysed by immunofluorescence for the junctional markers ZO-1 and β-catenin (**A**), p114RhoGEF and AKAP12 (**B**), or F-actin and Vinculin (**C**). Bars: 40µm.
